# Colonization of nasal cavities by *Staphylococcus epidermidis* mitigates SARS‐CoV‐2 nucleocapsid phosphoprotein‐induced interleukin (IL)‐6 in the lung

**DOI:** 10.1111/1751-7915.13994

**Published:** 2022-04-14

**Authors:** Ming‐Shan Kao, Jen‐Ho Yang, Arun Balasubramaniam, Supitchaya Traisaeng, Albert Jackson Yang, John Jackson Yang, Benjamin Prethiviraj Salamon, Deron R. Herr, Chun‐Ming Huang

**Affiliations:** ^1^ Department of Biomedical Sciences and Engineering National Central University Taoyuan 32001 Taiwan; ^2^ Department of Life Sciences National Central University Taoyuan 32001 Taiwan; ^3^ Department of Biology San Diego State University San Diego CA 92182 USA; ^4^ Department of Biomedical Science and Environment Biology Kaohsiung Medical University Kaohsiung 80708 Taiwan

## Abstract

Infection by severe acute respiratory syndrome coronavirus 2 (SARS‐CoV‐2) can trigger excessive interleukin (IL)‐6 signalling, leading to a myriad of biological effects including a cytokine storm that contributes to multiple organ failure in severe coronavirus disease 2019 (COVID‐19). Using a mouse model, we demonstrated that nasal inoculation of nucleocapsid phosphoprotein (NPP) of SARS‐CoV‐2 increased IL‐6 content in bronchoalveolar lavage fluid (BALF). Nasal administration of liquid coco‐caprylate/caprate (LCC) onto *Staphylococcus epidermidis* (*S. epidermidis*)‐colonized mice significantly attenuated NPP‐induced IL‐6. Furthermore, *S. epidermidis‐*mediated LCC fermentation to generate electricity and butyric acid that promoted bacterial colonization and activated free fatty acid receptor 2 (Ffar2) respectively. Inhibition of Ffar2 impeded the effect of *S. epidermidis* plus LCC on the reduction of NPP‐induced IL‐6. Collectively, these results suggest that nasal *S. epidermidis* is part of the first line of defence in ameliorating a cytokine storm induced by airway infection of SARS‐CoV‐2.

## Graphical abstract

Nasal administration of liquid coco‐caprylate/caprate (LCC) onto Staphylococcus epidermidis (S. epidermidis)‐colonized mice significantly attenuated NPP‐induced IL‐6. Inhibition of Ffar2 impeded the effect of S. epidermidis plus LCC on the reduction of NPP‐induced IL‐6. Collectively, these results suggest that nasal S. epidermidis is part of the first line of defense in ameliorating a cytokine storm induced by airway infection of SARS‐CoV‐2.

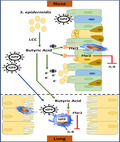

## Introduction

Severe coronavirus disease 2019 (COVID‐19)‐associated pneumonia patients develop features of systemic hyper‐inflammation under the umbrella terms of ‘macrophage activation syndrome’ or ‘cytokine storm’ (McGonagle *et al*., 2020). Pro‐inflammatory cytokines, especially interleukin (IL)‐6, are highly elevated in severely ill COVID‐19 patients (Chen *et al*., 2020; Huang *et al*., 2020a; Qin *et al*., 2020; Tan *et al*., 2020). Severe acute respiratory syndrome coronavirus 2 (SARS‐CoV‐2) selectively induces IL‐6 production and results in the exhaustion of lymphocytes (Tang *et al*., 2020). It has been documented that the nucleocapsid phosphoprotein (NPP) of SARS‐CoV‐2 is essential for viral activation of IL‐6 gene induction (Zhang *et al*., 2007). Furthermore, NPP of SARS‐CoV can directly bind to nuclear factor kappa‐light‐chain‐enhancer of activated B cells (NF‐κB) response elements and can activate IL‐6 expression by facilitating the translocation of NF‐κB from cytosol to nucleus (Zhang *et al*., 2007).

*Staphylococcus epidermidis* (*S. epidermidis*) is a Gram‐positive bacterium that is an abundant component of the microbiome of the human nasal mucosa and the respiratory tract. Several studies provide evidence for a protective role of nasal *S. epidermidis* in the defence against viral infection. For instance, the extracellular matrix‐binding protein (Embp) of *S. epidermidis* can bind directly to influenza virus (Chen *et al*., 2016) and intranasal inoculation of Embp induces the expression of antiviral cytokines in the nasal tissues. In addition, exposure of nasal epithelia cells to nasal‐derived *S. epidermidis* was shown to increase expression and release of interferon (IFN)‐λ in response to influenza virus exposure (Kim *et al*., 2019). Data from our laboratory has revealed probiotic activity of *S. epidermidis,* which can metabolize carbon‐rich molecules such as glycerol to yield short‐chain fatty acids (SCFAs) (Keshari *et al*., 2019; Traisaeng *et al*., 2019) and electrons (Balasubramaniam *et al*., 2020). The carbon‐rich molecules provided *S. epidermidis* with carbon sources or electron donors to generate the electricity through fermentation. It has been shown that electrogenic bacteria can mediate electron production to conserve energy to support growth (Sacco *et al*., 2017). Furthermore, the anti‐inflammatory properties of SCFAs such as butyric acid and propionic acid have been identified in the pathogen‐infected tissues (Kao *et al*., 2017). Cumulatively, these studies suggest that *S. epidermidis* in the nasal microbiome may act as a front‐line defender against virus infection. Suppression of early‐stage viral infection by nasal bacteria may reduce nasal invasion of SARS‐CoV‐2 and minimize airborne transmission of COVID‐19.

The challenges in developing an effective COVID‐19 vaccine included antigenic drift and increased risk of adverse effects due to fast‐tracked development. Furthermore, the cases of COVID‐19 reinfection have been recently identified (Lu *et al*., 2020), raising concerns regarding the durability of vaccine‐induced responses and the efficacy of this approach in developing herd immunity. Also, the low effectiveness against SARS‐CoV‐2 variants among COVID‐19 vaccinated persons has been reported (Dong *et al*., 2021; Levine‐Tiefenbrun *et al*., 2021). Antiviral drugs, such as remdesivir, have shown some promise in initial clinical trials, but may be ineffective if administered late in COVID‐19 progression. The use of nasal bacteria may offer a valuable therapeutic approach to combat SARS‐CoV‐2, in place of or in addition to vaccines and antiviral drugs. Such bacteria are natural inhabitants of the upper respiratory tract, their action does not require full host immune stimulation, and they can hinder viral entry at the early stages of infection. We evaluated this effect *in vivo* by applying liquid coco‐caprylate/caprate (LCC) to the nasal cavities of mice to trigger fermentation of resident *S. epidermidis*. LCC, a Food and Drug Administration (FDA)‐approved compound with C8‐10 fatty acid connected to C12‐C18 fatty alcohols, is currently used as a topical emollient to minimize ultraviolet (UV) radiation exposure (Sohn *et al*., 2020). Our results identify the novel biological function of *S. epidermidis* as a nasal commensal bacterium capable of protecting against SARS‐CoV‐2. We further provide evidence for the potential value of LCC as a repurposed or repositioned drug (Beigel *et al*., 2020; Tomazini *et al*., 2020) against COVID‐19.

## Results

### Nasal inoculation of NPP of SARS‐CoV‐2 in mice induces an increase in IL‐6 in the lung

**Fig. 1:**
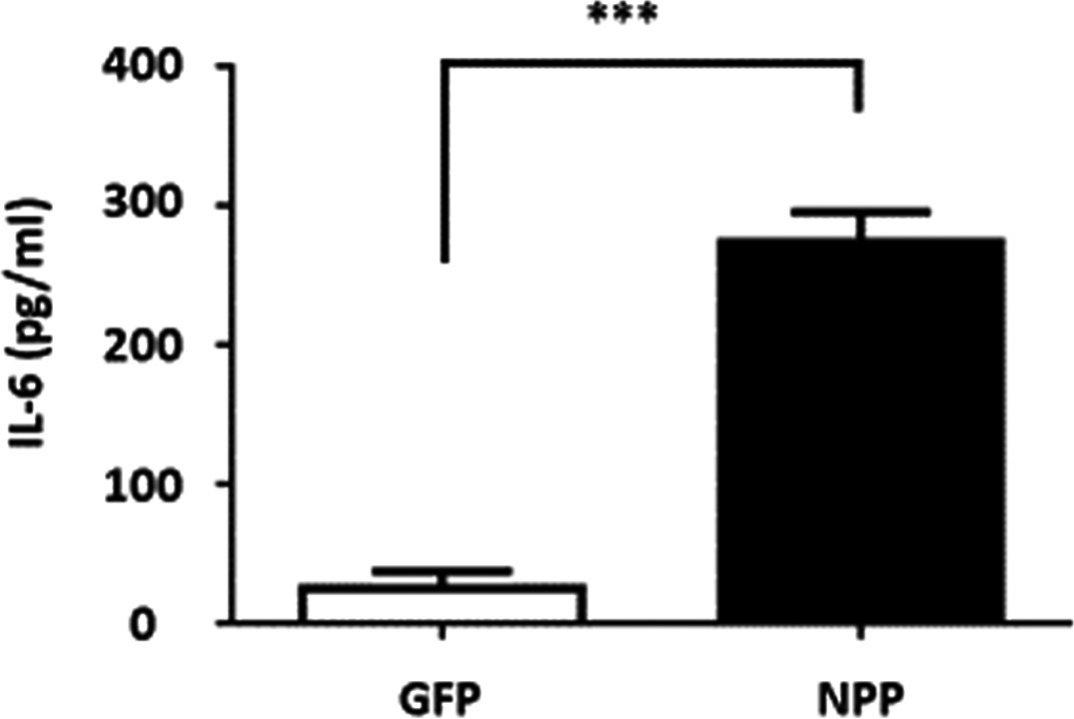
Nasal inoculation of recombinant NPP increased IL‐6 in BALF in mice (A) Recombinant NPP or GFP was inoculated into the nasal cavities of ICR mice for 6 h. The levels of IL‐6 in BALF were measured by ELISA. The level of IL‐6 induced by recombinant NPP was expressed as fold change over that induced by recombinant GFP. The *P*‐value of < 0.001 (***) from three different experiments with mean ± SD was shown.

The gene‐encoding NPP [protein identification (ID): YP_009724397.2] of SARS‐CoV‐2 was transformed into *Escherichia coli* (*E. coli*) BL21 competent cells. After isopropyl β‐D‐thiogalactopyranoside (IPTG) induction and purification, recombinant NPP with an apparent molecular mass of approximately 48 kDa determined by sodium dodecyl sulphate‐polyacrylamide gel electrophoresis (SDS‐PAGE) was obtained. Expression of recombinant green fluorescent protein (GFP) in *E. coli* BL21 competent cells was conducted by following the same procedure. IL‐6 plays a central role in both innate and adaptive immunities and is a key component of the cytokine storm during the progression of COVID‐19. Up‐regulation of IL‐6 can be induced by infection of many viruses including SARS‐CoV (Wang *et al*., 2007). To examine whether NPP of SARS‐CoV‐2 can regulate the IL‐6 production, we intranasally inoculated recombinant NPP or control GFP into Institute of Cancer Research (ICR) mice for 6 h. The level (273.90 ± 8.86 pg ml^−1^) of IL‐6 in bronchoalveolar lavage fluid (BALF) collected from NPP‐inoculated mice was approximately 10‐fold higher than that (25.03 ± 4.98 pg ml^−1^) in BALF collected from GFP‐inoculated mice (Fig. 1A). This result demonstrates for the first time that NPP of SARS‐CoV‐2 is sufficient to up‐regulate IL‐6 in the lung and supported the previous finding that NPP of other coronavirus can activate IL‐6 gene expression by binding directly to NF‐κB regulatory element on IL‐6 promoter (Zhang *et al*., 2007).

### LCC fermentation mediates electricity production that is essential for nasal colonization of S. epidermidis

**Fig. 2:**
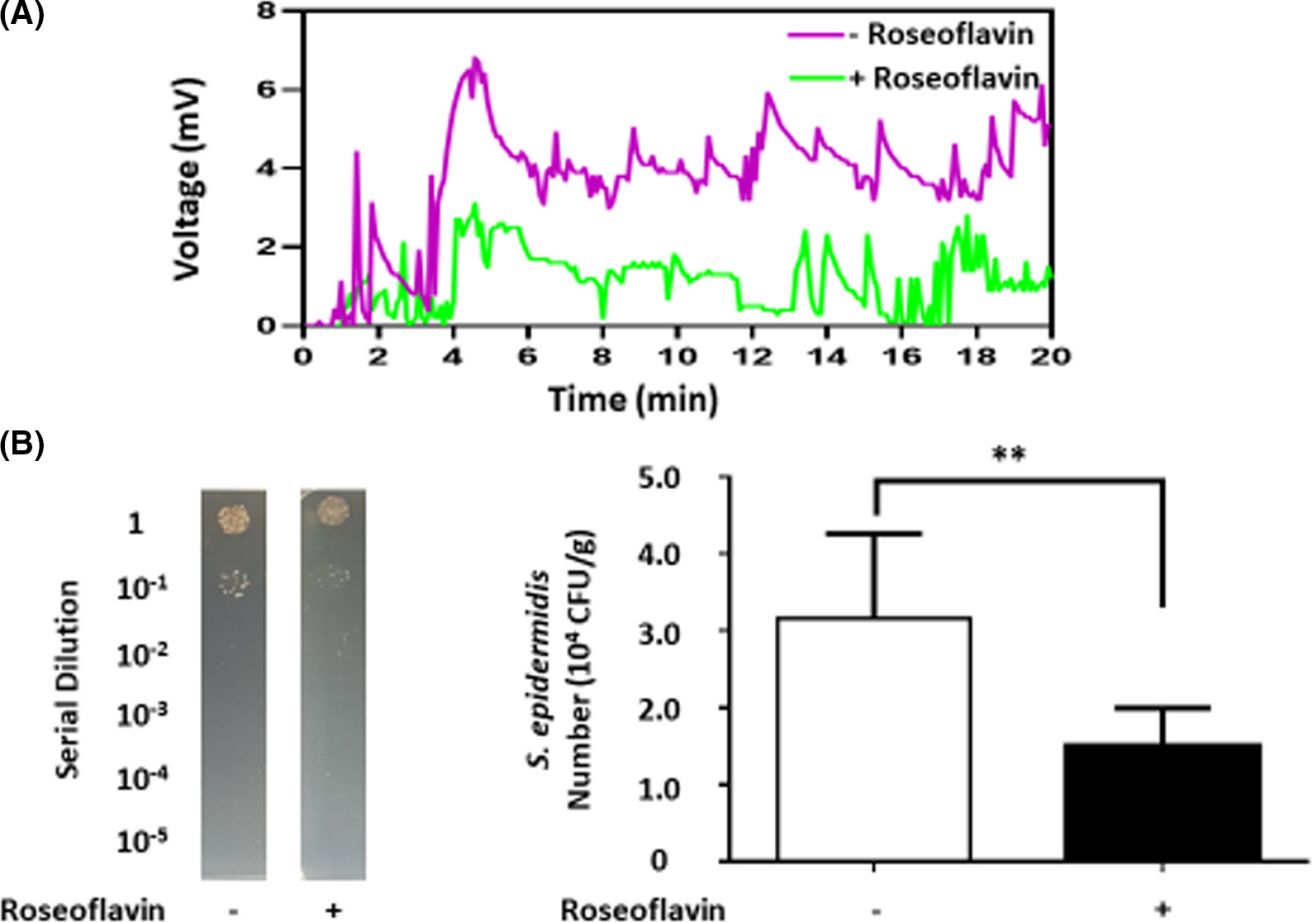
Bacterial electricity and nasal colonized were reduced by pretreatment of *S. epidermidis* with roseoflavin (A) Electricity indicated by voltage changes (mV) was detected in the LCC culture of *S. epidermidis* pretreated with/without roseoflavin. (B) Nasal cavities of ICR mice were inoculated daily with *S. epidermidis* pretreated with/without roseoflavin for 3 days. The number (CFU g^−1^) of *S. epidermidis* on nasal cavities were counted by plating serial dilutions (1:10^0^–1:10^5^) of nasal homogenates on a streptomycin supplemented TSB agar plate. Bacterial CFU per gram (CFU g^−1^) of excised nasal cavities was calculated. Data are represented as mean ± SD with the *P*‐value of < 0.01 (**) from experiments in triplicate. Five mice per group were evaluated.

Data from our previous analysis have revealed that *S. epidermidis* fermentatively metabolizes glycerol (C_3_H_8_O_3_) as a carbon source to yield electricity (Balasubramaniam *et al*., 2020). The process of generating electrons in *S. epidermidis* was similar to flavin‐based extracellular electron transfer (EET) in other Gram‐positive bacteria (Light *et al*., 2018). Riboflavin kinase (EC 2.7.1.26) is a key enzyme that catalyses the synthesis of flavin mononucleotide (FMN) from riboflavin (Sebastian *et al*., 2019). Roseoflavin, as an inhibitor of riboflavin kinase, can block bacterial riboflavin riboswitches (Miroux and Walker, 1996; Mansjo and Johansson, 2011). Here, *S. epidermidis* without pretreatment of roseoflavin was placed on the anode of a digital multimetre in the presence of LCC with a longer carbon chain (C8‐10 fatty acid connected to C12‐C18 fatty alcohols). As shown in Fig. 2A, a significant increase in voltage with a peak value of approximately 6 mV was observed throughout the 20 min monitoring period when media contained *S. epidermidis* plus LCC. The voltage change provoked by *S. epidermidis* in the presence of LCC was completely attenuated when *S. epidermidis* was pretreated with roseoflavin, indicating that riboflavin kinase was involved in flavin‐based EET in electrogenic *S. epidermidis*.

The bacterial EET system involves bacterial communication and attachment architecturally complex microbial communities such as biofilm (Patil *et al*., 2012; Sarjit *et al*., 2015). We thus evaluated whether electricity produced by *S. epidermidis* plus LCC influences the bacterial nasal colonization. *S. epidermidis* pretreated with or without roseoflavin was intranasally inoculated onto mice every day for three days. Bacterial residence in the nasal cavity was quantified by culturing limiting dilutions of tissue homogenates on streptomycin‐supplemented tryptic soy broth (TSB) agar plates. Results demonstrated that only inoculated *S. epidermidis*, but not endogenous bacteria, can grow colonies on streptomycin supplemented TSB agar plates (Fig. S1). By counting bacterial colony forming unit (CFU) in nasal homogenates after three days of inoculation, we found that the *S. epidermidis* content in mice pretreated with roseoflavin (1.50 ± 0.20 × 10^4^ CFU g^−1^) was approximately half that of mice that did not receive roseoflavin pretreatment (3.16 ± 0.49 × 10^4^ CFU g^−1^) (Fig. 2B). These results suggest that riboflavin kinase‐mediated electricity production of *S. epidermidis* may affect bacterial colonization of the nasal sinus.

### Produced of butyric acid by S. epidermidis‐colonized nasal septum

**Fig. 3:**
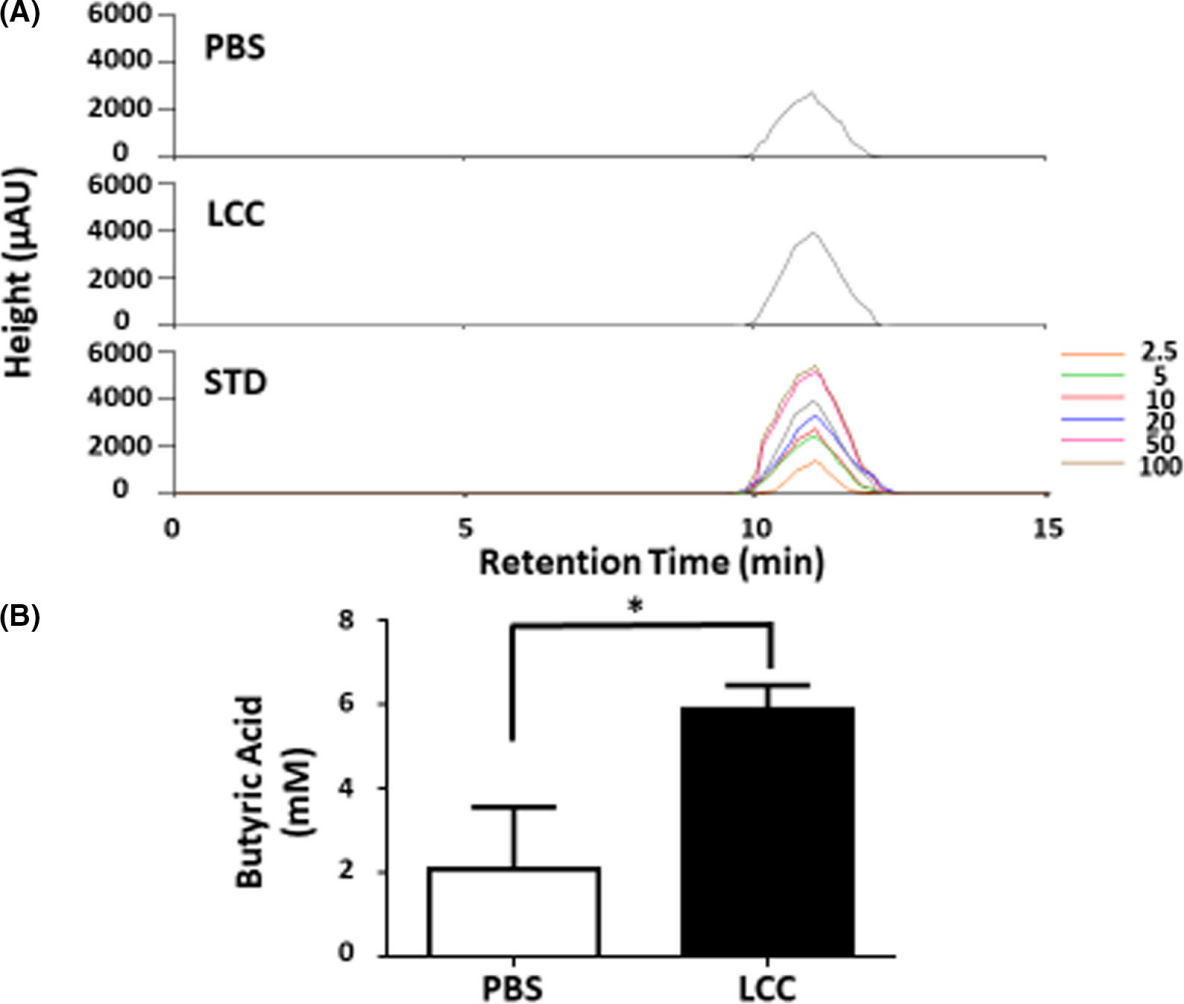
LCC induced production of butyric acid of *S. epidermidis* colonized on nasal septum (A) The epithelial layer of isolated nasal septum was placed with *S. epidermidis* with or without LCC in rich media for 24 h. Representative chromatograms of HPLC analysis of butyric acid in media were shown. (B) The concentration (mM) of butyric acid was quantified based on the heights [milli‐absorbance unit (mAU)] of standard (STD) peaks with concentrations of butyric acid from 0‐100 mM. Data are represented as mean ± SD. The *P*‐value of < 0.001 (***) was obtained from three different experiments.

*S. epidermidis* can mediate fermentation to produce SCFAs. Butyric acid, one of SCFAs, functions as an electron donor (Finke *et al*., 2007) and has been shown to exert potent anti‐inflammatory effects (Keshari *et al*., 2020). To investigate if *S. epidermidis* can metabolize LCC to produce butyric acid, the nasal septa isolated from ICR mice were topically applied with *S. epidermidis* in the presence of 2% LCC and incubated in rich media for 24 h. Incubation of nasal septa with *S. epidermidis* in the absence of LCC served as a control. High‐performance liquid chromatography (HPLC) analysis was conducted to quantify the level of butyric acid in media of bacteria. The concentration of butyric acid in media conditioned by LCC‐treated bacteria on nasal septa was markedly higher than that in media from control cultures (Fig. 3). The different concentrations of butyric acid (0–100 mM) were subjected to HPLC to establish a quantitative standard curve. As shown in Fig. 3B, LCC fermentation of *S. epidermidis* on nasal septum for 24 h yielded approximately 5.87 mM of butyric acid.

### *S. epidermidis* plus LCC attenuates NPP‐induced IL‐6 production in BALF via activation of free fatty acid receptor 2 (Ffar2)

**Fig. 4:**
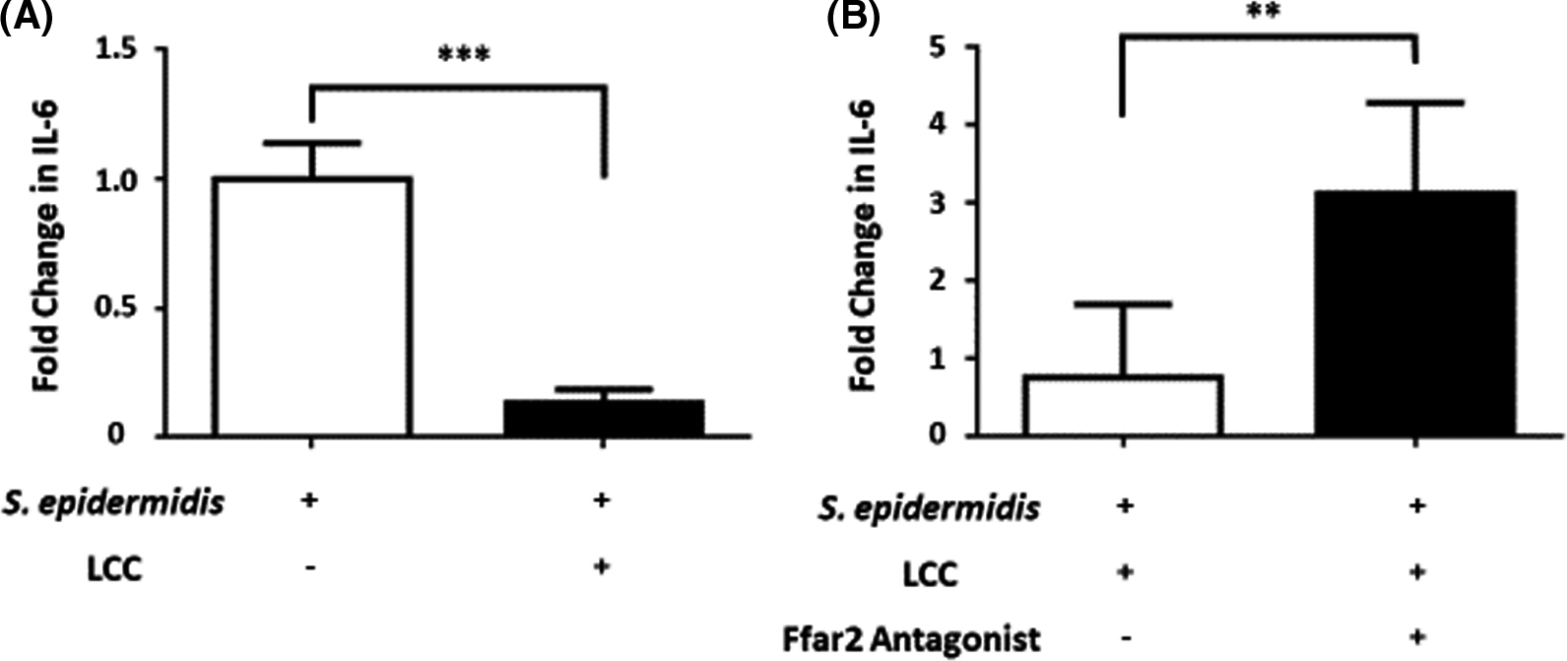
Involvement of Ffar2 in the reduction of NPP‐induced IL‐6 by inoculation of LCC onto *S. epidermidis*‐colonized mice (A) *S. epidermidis*‐colonized ICR mice were intranasally applied with LCC (+LCC) or PBS (‐LCC) 1 h before inoculation of NPP for 6 h. The level of IL‐6 in BALF induced by NPP was expressed as fold change over that induced by PBS. (B) *S. epidermidis*‐colonized ICR mice were given GLPG‐0974, a Ffar2 antagonist (+), or vehicle (ethanol) alone (‐) by gastric gavage every day for 3 days. After administration of GLPG‐0974, mice were intranasally applied with LCC six hours before inoculation with NPP. The level of IL‐6 in BALF of mice given with GLPG‐0974 was expressed as fold change over that in BALF of mice given without GLPG‐0974. The *P*‐value of < 0.001 (**); < 0.0001 (***) from three independent experiments with mean ± SD is shown.

To assess whether butyric acid (Fig. 3) and electron (Fig. 2) production can regulate NPP‐induced IL‐6, LCC or phosphate‐buffered saline (PBS) (control) were intranasally administered into the mice nasally colonized with *S. epidermidis* K1, a strain isolated from human nasal cavities (Table S1) before inoculation with recombinant NPP. The level of IL‐6 in BALF was quantified by sandwich enzyme‐linked immunosorbent assay (ELISA) 6 h after NPP inoculation. Compared with PBS control, the administration of LCC significantly reduced NPP‐induced IL‐6 in BALF (Fig. 4A). Administration of LCC into nasal cavities of mice without *S. epidermidis* colonization did not alter the level of IL‐6 in BALF compared with administration of water (Fig. S2). These results indicated that LCC alone had no effect on reduction of IL‐6 induced by NPP, but rather, is likely to be metabolized by *S. epidermidis* fermentation into anti‐inflammatory butyric acid.

Ffar2, also known as G protein‐coupled receptor 43 (GPR43), is expressed by various cells including macrophages and epithelial cells in nasal cavities and lung (Wang *et al*., 2020). Butyric acid is a potent (micromolar) agonist of Ffar2. To validate the contribution of Ffar2 to the effect of butyric acid‐producing *S. epidermidis* on lowering NPP‐induced IL‐6, GLPG‐0974 was given to *S*. *epidermidis*‐colonized mice to antagonize the Ffar2 before administration of LCC. Compared with IL‐6 content (132.0 ± 56.54 pg ml^−1^) in mice treated with vehicle, treatment with GLPG‐0974 resulted in a nearly 3‐fold increase in the level (442.0 ± 69.95 pg ml^−1^) of IL‐6 in BALF (Fig. 4B). In the macrophage culture experiment, IL‐6 level also increased 2 times after treated with GLPG‐0974 in the media containing butyric acid (Fig. S3). Taken together, these data demonstrate that LCC fermentation of nasal *S. epidermidis* requires Ffar2 for its protective effect in attenuating NPP‐induced IL‐6 production.

## Discussion

Previous studies have revealed that NPP (Sequence ID: AAR12990.1) of SARS‐CoV (SARS coronavirus HB) can bind directly to the NF‐κB recognition elements on the IL‐6 promoter and regulate IL‐6 expression (Zhang *et al*., 2007). The region from amino acids 86 to 96 (GYYRRATRRAR) at the N terminus of SARS‐CoV NPP is essential for the viral protein to activate IL‐6 transcription. The NPP of SARS‐CoV‐2 in this study shares 90% sequence identity to NPP (Sequence ID: AAR12990.1) of SARS‐CoV. Furthermore, amino acids 85 to 95 (GYYRRATRRIR) at the N terminus of SARS‐CoV‐2 NPP differ by only one amino acid compared with SARS‐CoV NPP. This amino acid alignment suggests that the increase in IL‐6 after nasal inoculation of SARS‐CoV‐2 NPP (Fig. 1B) may be due to the binding of NPP to NF‐κB response elements in cells of the nose and lung. To ensure the purified NPP could trigger the inflammation in the cells, the treatment of macrophage experiment was done and we found purified NPP induced approximately 4 times IL‐6 higher than GFP (Fig. S3). The results also supported the utility of targeting the IL‐6 pathway for the treatment of COVID‐19. Although three neutralizing antibodies that target IL‐6 signalling are being evaluated for efficacy as COVID‐19 therapeutics (Solis‐Garcia Del Pozo *et al*., 2020), we took an approach of drug repurposing to validate the effect of nasal inoculation of LCC on NPP‐induced IL‐6. Although both NPP and GFP purified from *E. coli* BL21 may contain lipopolysaccharide (LPS), the secretion of IL‐6 induced by NPP was approximately 10‐fold higher than that induced by GFP (Fig. 1A), demonstrating that the IL‐6 secretion is mainly triggered by NPP, not LPS.

LCC is an ester synthesized from the reaction of the coconut alcohol‐derived fatty acids with a mixture of caprylic acid and capric acid (Fiume *et al*., 2015). Literature has reported that caprylic acid or capric acid exert anti‐inflammatory activity via down‐regulation of NF‐κB (Huang *et al*., 2014; Zhang *et al*., 2019). However, our results indicate that nasal administration of LCC did not directly affect NPP‐induced IL‐6 in BALF (Fig. S2). One possible explanation is that LCC, as a complex of medium chain fatty acids, may need to be catabolized before entering cells to down‐regulate the NF‐κB. We show that *S. epidermidis* can fermentatively metabolize LCC to butyric acid and yield electricity (Figs. 2 and 3). Butyric acid can inhibit histone deacetylase to reduce lung inflammation by regulating the Toll‐like receptor/NF‐κB signalling pathway (Liu *et al*., 2019). We further show that reduction of NPP‐induced IL‐6 in BALF by nasal administration of LCC onto *S. epidermidis* colonized mice is mediated by Ffar2 (Fig. 4), since *S. epidermidis*‐colonized mice orally given GLPG‐0974, a Ffar2 antagonist, retained elevated levels of NPP‐induced IL‐6 in BALF after nasal administration of LCC. A pharmacokinetic and pharmacodynamic study revealed that GLPG‐0974 was detectable in the blood and inactivated Ffar2 of neutrophils in the blood (Namour *et al*., 2016). Thus, when mice were given GLPG‐0974 via oral gavage, GLPG‐0974 can be delivered to the upper respiratory tract to inhibit Ffar2 expressed by immune cells and epithelia cells of nose and lung. It has been shown that SCFAs can activate Ffar2 to ameliorate inflammation initiated by NF‐κB signalling (Huang *et al*., 2020b). Thus, we envision that *S. epidermidis*‐colonized nasal cavities can convert LCC to butyric acid and electrons. Butyric acid may bind to Ffar2 in cells in the upper respiratory tract to down‐regulate NF‐κB and IL‐6 secretion. Angiotensin‐converting enzyme 2 (ACE‐2) has been identified as a SARS‐CoV‐2 receptor (Gilzad‐Kohan and Jamali, 2020), and our current work demonstrates that Ffar2 attenuates SARS‐CoV‐2 pathology. Future work will investigate whether Ffar2 regulates inflammation after SARS‐CoV‐2 binds to ACE‐2.

Bacteria can excrete electron acceptors such as flavin molecules that act as small diffusible shuttle molecules to transfer electrons (Marsili *et al*., 2008). We show here that inhibition of riboflavin kinase by 1 μM roseoflavin markedly attenuates electricity production of *S. epidermidis* plus LCC (Fig. 2A). Although a direct anti‐bacterial activity of roseoflavin has been reported (Lee *et al*., 2009), we found that incubation of *S. epidermidis* (10^7^ CFU) with roseoflavin at concentrations less than 1 μM for 24 h did not affect the formation of bacterial colonies on TSB agar plates (data not shown). Thus, inhibition of bacterial electricity production by pretreatment of *S. epidermidis* with roseoflavin may be due to a decrease in the synthesis of flavin molecules, rather than the inhibition of bacterial growth. The different direct electric currents can affect bacterial surface properties and adhesion (Luo *et al*., 2005). Bacterial aggregation can be promoted by adding electron acceptors (Gomez‐Carretero *et al*., 2017). Furthermore, bacteria can use SCFAs as electron donors and oxygen near the epithelial cells of host as an electron acceptor to generate a proton motive force that sustains efficient motility and growth for bacterial colonization (van der Stel *et al*., 2017). As shown in Fig. 2, roseoflavin‐pretreated *S. epidermidis* has significantly lost the ability to yield electricity and colonize nasal cavities of mice. *S. epidermidis* may utilize LCC to produce butyric acid as an electron donor and FMN as an electron acceptor. Oxygen on the surface of nasal epithelial cells also acts as an electron acceptor. The electron transfer between donors (butyric acid) and acceptors (FMN and oxygen) may facilitate bacterial colonization. Roseoflavin treatment may reduce the FMN production (Lee *et al*., 2009) and disrupt electron transfers, leading to poor colonization of *S. epidermidis* on nasal cavities.

To investigate whether wild‐type *S. epidermidis* from human nostrils can undergo LCC fermentation as efficiently as *S. epidermidis* ATCC 12228, a non‐biofilm forming laboratory (MacLea and Trachtenberg, 2017), nasal swabs were collected from one healthy subject and spread on TSB agar plates. Eleven colonies on TSB agar plates were selected for 16S rRNA sequencing. Seven of eleven colonies were identified as *S. epidermidis* (K1, K2, H1, H2, 2, 4 and 5 strains) with 99.6–100% sequence identity to *S. epidermidis* ATCC 12228 (Table S1). Four colonies were identified as *Bacillus pumilus* (*B. pumilus*), *Terribacillus goriensis* (*T. goriensis*), *Kocuria indica* (*K. indica*) and *Corynebacterium* segmentosum (*C. segmentosum*). To examine the fermentative capabilities, *S. epidermidis* K1 strain was cultured in rich media in the presence of 2% LCC for 12 h. Rich media with LCC alone or *S. epidermidis* K1 alone served as controls. The media in the culture of *S. epidermidis* K1 with LCC turned yellow after incubation for 12 h, while the media in the other three conditions maintained their original colours (Fig. S4A), indicating that *S. epidermidis* K1 strain has a capability of fermenting LCC. To investigate the bacterial electrogenicity, *S. epidermidis* K1 strain with 2% LCC in rich media was added onto the surface of the anode. Addition of the same volume of media, *S. epidermidis* K1 alone, or 2% LCC alone acted as controls. As shown in Fig. S4B, little or no voltage change was detected in media alone or media containing LCC alone. The approximately 4 mV current detected in rich media containing *S. epidermidis* K1 alone could be because bacteria can utilize dextrose in TSB of rich media as an electron donor. However, a marked increase in voltage to 6 mV was detected when 2% LCC was added to media containing *S. epidermidis* K1. In addition, by performing the quantitative real‐time polymerase chain reaction (qPCR), we found that the expression of genes, pyruvate dehydrogenase (*pdh*) and type II NADH: quinone oxidoreductase (*ndh2*), related to EET in *S. epidermidis* K1, was higher than that in *S. epidermidis* ATCC 12228 (Fig. S5). These results support that the human nasal *S. epidermidis* K1 can undergo LCC fermentation and produce electricity.

It has been reported that the abundance of *Fusobacterium periodonticum* (*F. periodonticum*), a bacterium in the human nasopharyngeal microbiome, significantly decreased after SARS‐CoV‐2 infection (Moore *et al*., 2020; Nardelli *et al*., 2021). The sialic acid residues on the surface of *F. periodonticum* worked as alternative receptors of the SARS‐CoV‐2 spike protein, decreasing the probability of viral binding to human ACE‐2 positive nasal epithelial cells, the main target of invasion for SARS‐CoV‐2 to enter into the human body (Morniroli *et al*., 2020; Gallo *et al*., 2021). Previous studies demonstrated that the nasal *S. epidermidis* significantly reduced the expression of ACE‐2 gene in host cells (Ji *et al*., 2021). These results revealed the beneficial effects of nasal commensal bacteria on reduction of SARS‐CoV‐2 infection.

In summary, immune responses activated by SARS‐CoV‐2 infection lead to uncontrolled inflammation including IL‐6 release and ultimately resulting in a cytokine storm (Cao, 2020). Our data have demonstrated for the first time that, when given LCC, nasal *S. epidermidis* carries out fermentation to yield butyric acid as well as electrons. This electron production may facilitate bacterial nasal colonization, while the butyric acid can activate Ffar2 to down‐regulate NPP‐induced IL‐6 production. These findings provide evidence that *S. epidermidis* colonization in the upper airway in humans can be considered the respiratory system's first line of defence against SARS‐CoV‐2 infection.

## Experimental procedures

### Ethics statement

Mouse experiments were performed in accordance with a protocol (NCU‐106‐016, 19 December 2017) of the Institutional Animal Care and Use Committee (IACUC) of National Central University (NCU). Female ICR mice (8–9 weeks old) were obtained from the National Laboratory Animal Center Taipei, Taiwan. Mice were sacrificed by CO_2_ asphyxiation under isoflurane anaesthesia in an encased chamber.

### Sources of nasal swabs

Nasal swabs were collected from a healthy human subject. Written consent was obtained from the participant prior to inclusion in the study. The Institutional Review Board (IRB) (No. 19‐013‐B1, 22 May 2019) at Landseed International Hospital, Taiwan, approved the consent procedure for nasal swab sampling.

### NPP cloning, expression and purification

A plasmid carrying a gene encoding NPP (ID: YP_009724397.2) of SARS‐CoV‐2 was transformed into *E. coli* BL21 competent cells (Yeastern Biotech Co., Ltd, Taiwan). The *E. coli* BL21 transformed with a plasmid encoding GFP was used as a control. The *E. coli* BL21 was cultivated in 1 L Luria‐Bertani (LB) broth (Becton, Dickinson and Company, Franklin Lakes, NJ, USA) supplemented with 50 μg ml^−1^ kanamycin at 37°C. At optical density OD_600nm_ = 0.6, 1 mM of IPTG was added into the culture of *E. coli* BL21 at 20°C for 16 h to induce protein expression. Proteins were purified by the ProBond^TM^ Purification System (Invitrogen, Carlsbad, CA, USA) and visualized by Coomassie Brilliant Blue stained gels of 10% SDS*‐*PAGE.

### Nasal inoculation and IL‐6 detection

Recombinant NPP (50 μg in 20 μl of PBS) was inoculated to the nasal cavities of ICR mice. Inoculation with the same amount of recombinant GFP served as a control. Six hours after inoculation, the tracheas in anesthetized mice were cannulated with a catheter. The BALF was obtained by lavaging the lungs 3 times each with 1‐ml instillation (3 ml total) of PBS. The total lavage fluid from each mouse lung was pooled and centrifuged at 2000 rpm for 10min. The level of IL‐6 in the supernatant was quantified by ELISA using a Quantikine mouse IL‐6 set (R&D Systems, Minneapolis, MN, USA). The level of IL‐6 induced by NPP was normalized to that induced by GFP. Results were expressed as fold change over the GFP control.

### Bacterial electricity detection

*S. epidermidis* ATCC 12228 was cultured in TSB (Sigma) at 37°C. The bacterial pellet was harvested by centrifugation at 15 000 *g* for 5 min, suspended in PBS. Electricity produced by *S. epidermidis* was measured by the changes in voltage (mV) against time (min) using a digital multimeter (Lutron, DM‐9962SD, Sydney, Australia). Voltages were recorded every 5 s using a device equipped with a carbon felt (2.5 cm × 10 cm) and a carbon cloth (10 cm × 10 cm) (Homy Tech, Taoyuan, Taiwan) as anode and cathode respectively. The cathode was covered with a Nafion membrane N117 (6 cm × 6 cm) (Homy Tech) as a proton exchange membrane (PEM). Copper wires were used to link the anode and cathode to an external resistance (1000 Ω). *S. epidermidis* pretreated with or without 1 μM of roseoflavin (Sigma) for 24 h in the presence of LCC (TNJC corporation, Chiayi, Taiwan) was pipetted on the surface of the anode.

### Isolation of nasal septum and detection of butyric acid by HPLC

Mouse nasal septum was isolated according to a protocol as previously described (Antunes *et al*., 2007). The isolated nasal septum with epithelium facing up was placed in 500 μl rich media [10 g l^−1^ yeast extract (Biokar Diagnostics, Beauvais, France), 5 g l^−1^ TSB, 2.5 g l^−1^ K_2_HPO_4_ and 1.5 g l^−1^ KH_2_PO_4_]. *S. epidermidis* ATCC 12228 (10^8^ CFU) in the absence or presence of 2% LCC was pipetted on the top of mouse nasal septum for 24 h. To detect butyric acid, media were collected and filtered through a 0.22 μm microfiltration membrane to remove bacteria and insoluble particles. One hundred microlitres of concentrated hydrochloric acid were added before samples were extracted for 20 min by gently rolling using 5 ml of diethyl ether. After centrifugation at 1100 *g* for 5 min, the supernatant was transferred to another Pyrex extraction tube before 500 μl of a 1‐mM solution of NaOH was added. The aqueous phase was moved to an autosampler vial and 100 μl of concentrated hydrochloric acid were added. The analysis of butyric acid was conducted using an Agilent 1200 series HPLC system with a ZORBAX Eclipse XDB‐C18 column (4.6 × 250 mm, 5 μm). The mobile phase was made up of 20 mmol l^−1^ NaH_2_PO_4_ solution (pH 2.2) and acetonitrile. The detector was set at 210 nm. The concentration of butyric acid was calculated based on a calibration curve of a butyric acid analytical standard.

### Colonization of *S. epidermidis* on mouse nasal cavities

To colonize *S. epidermidis* on mouse nasal cavities, ICR mice were intranasally inoculated 10^8^ CFU/ 20 µl *S. epidermidis* every day. Three days after inoculation, nasal cavities were excised and homogenized in sterile PBS (0.1 g/100 μl). The CFUs of inoculated *S. epidermidis* on nasal cavities were enumerated by plating serial dilutions (1:10^0^–1:10^5^) of homogenates on a 500 µg ml^−1^ streptomycin supplemented TSB agar plate at 37°C for 24 h. For some experiments, mouse nasal cavities without inoculation of *S. epidermidis* were homogenized and plated on streptomycin‐supplemented TSB agar plates to count the endogenous bacteria on mouse nasal cavities. Sequence analysis of 16S ribosomal RNA (rRNA) genes was utilized for bacterial identification. A single colony of bacteria from a TSB agar plate was isolated with a sterile toothpick and boiled at 100°C for DNA extraction using an Easy Pure Genomic DNA Spin Kit (Bioman, New Taipei, Taiwan). Identification of *S. epidermidis* was confirmed by 16S rRNA sequencing using the 16S rRNA 27F and 534R primers for PCR (Wang *et al*., 2014). The 16S rRNA gene sequences were analysed using the basic local alignment search tool (BLASTN, National Library of Medicine 8600 Rockville Pike, Bethesda, MD, USA).

### Inoculation of NPP into *S. epidermidis*‐colonized mice and inhibition of Ffar2

Mice colonized by *S. epidermidis* in nasal cavities were intranasally applied with 2% LCC or PBS 1 h before inoculation of recombinant NPP (50 μg in 20 μl PBS) for 6 h. The level of IL‐6 in BALF was 84.84 ± 14.84 pg ml^−1^ 6 h after nasal inoculation of PBS. For Ffar2 inhibition, GLPG‐0974, a Ffar2 antagonist (Tocris Bioscience, Bristol, UK), was dissolved in ethanol to make a stock solution. GLPG‐0974 (1 mg kg^−1^ body weight) was diluted in saline and then was given at 1 ml kg^−1^ body weight every day by gastric gavage for 3 days before LCC inoculation. Ethanol (50% in saline) was used as a vehicle control. Six hours after inoculation of recombinant NPP, the level of IL‐6 in BALF was measured by ELISA.

### Statistical analysis

GraphPad Prism^®^ software was applied for data analysis by unpaired t‐test. The statistical significance was determined by *P‐*values as follows: *P*‐values of < 0.05 (*), < 0.01 (**), and < 0.001 (***). The mean ± standard deviation (SD) was calculated from results obtained from at least three separate experiments.

## Limitations of the study

In this study, we have demonstrated that inoculation of LCC onto *S. epidermidis*‐colonized nasal cavities mitigated SARS‐CoV‐2 NPP‐induced IL‐6 content in BALF in mice. Although data *in vitro* and *ex vivo* showed that LCC triggered *S. epidermidis* fermentation and produced butyric acid and electricity, further in‐depth characterization of mechanism underlying the action of *S. epidermidis* fermentation to reduce IL‐6 *in vivo* would be pertinent.

## Resource availability

### Lead contact

Further information and requests for resources should be directed to and will be fulfilled by the lead Contact, Huang Chun‐Ming (chunming@ncu.edu.tw).

### Materials availability

All unique/stable reagents generated in this study are available, on reasonable request, from the Lead Contact on a completed Materials Transfer Agreement.

### Data and code availability

All data supporting the findings of this study are included in the article and its Supplemental Information or are available from the corresponding authors on request.

## Conflict of interest

The authors declare that there no conflicts of interest.

## Author contributions

Conceptualization: C.‐M.H. Methodology and Investigation: M.‐S.K., A.J.Y., J.J.Y., A.B. Resources: C.‐M.H. Writing‐Original draft: C.‐M.H., A.B., S.T., D.R.H. All authors read and provided input on the manuscript. Writing‐Review and Editing: C.‐M.H., M.‐S.K., A.B, S.T., A.J.Y. J.J.Y. B.P.S. Supervision and Project Administration: C.‐M.H. Funding Acquisition: C.‐M.H.

## Supporting information


**Fig. S1**. Nasal colonization of inoculated *S. epidermidis* was verified by 16S rRNA gene sequencing (A) *S. epidermidis* bacteria (ATCC 12228) (*S. epi*) (10^8^ CFU) or nasal homogenates (0.2 g) (NH) of ICR mice were serially diluted (1:10^0^‐1:10^5^) and placed on a 500 µg/mL streptomycin supplemented TSB agar plate at 37°C for 24 h. *S. epidermidis*, but not endogenous bacteria in nasal cavities, can grow on streptomycin supplemented TSB agar plates. (B) ICR mice were intranasally inoculated 10^8^ CFU/ 20 µL *S. epidermidis* every day for 3 days. Nasal homogenates (0.1 g/ 100 μL) were placed on a streptomycin supplemented TSB agar plate for 24 h. One of colonies (arrow) were selected for bacterial identification by 16S rRNA gene sequencing. (C) The nucleotide sequence of 16S rRNA gene of the selected colony shared 99% identity to *S. epidermidis* CLC‐M16.
**Fig. S2**. Nasal administration of LCC did not affect the NPP‐induced IL‐6. LCC (+LCC) or PBS (‐LCC) was administered to the nasal cavities of ICR mice 6 h before subsequent inoculation of recombinant NPP. The level of IL‐6 in BALF was measured by ELISA 6 h after NPP inoculation. The level of IL‐6 induced by LCC relative to that induced by PBS was expressed as fold change. Data are represented as mean ± SD, in triplicate, two‐tailed t‐tests. ns = non‐significant.
**Fig. S3**. Treatment of mouse macrophage J774A.1 cells with recombinant NPP induced a higher IL‐6 level compared to treatment of cells with recombinant GFP. The NPP‐induced of IL‐6 was reduced by pre‐treatment of cells with butyric acid. The reduction was partially restored by treatment of GLPG‐0974, a Ffar2 antagonist. ELISA was used to quantify IL‐6. Dara represented as mean ± SD, in triplicate, two‐tailed t‐tests. *P*‐values of <0.001 (***).
**Fig. S4**. A *S. epidermidis* strain isolated from human nasal cavities fermented LCC and produced electricity (A) A *S. epidermidis* K1 strain (10^7^ CFU) isolated from human nasal cavities (Table S1) was incubated in rich media (M) with/without LCC for 12 h. Rich media alone and rich media plus LCC without *S. epidermidis* were included as controls. The pH values of media 12 h after fermentation were indicated. The color of phenol red in rich media changed from red‐orange to yellow and reduction of pH values were used as indicators of bacterial fermentation. (B) The *S. epidermidis* K1 strain (10^7^ CFU) with/without 2% LCC in rich media was pipetted on an anode. Pipetting media alone (M) or LCC alone acted as a control. The voltage difference (mV) between anode and cathode was monitored for 20 min.
**Fig. S5**. The expression levels of genes (*pdh* and *ndh2*) in the *S. epidermidis* ATCC 12228 and K1 strains. RT‐qPCR was used to examine the relative expression of the *pdh* and *ndh2* genes which were normalized to 16S rRNA gene. Data shown represent the mean ± SD of experiments performed in triplicate, two‐tailed t‐tests. *P*‐values of <0.001 (***).
**Table S1**. 16S rRNA gene sequences of 11 bacterial colonies (K1, K2, H1, H2, and 1‐7) isolated from human nasal cavities.
**Table S2**. The designed primers for EET related genes.
