# Supplementary material for: Colonization of nasal cavities by Staphylococcus epidermidis mitigates SARS‐CoV‐2 nucleocapsid phosphoprotein‐induced interleukin (IL)‐6 in the lung
Source: Microb Biotechnol. 2022 Apr 14;15(7):1984–94. doi: 10.1111/1751-7915.13994 (PMC9111282; doi:10.1111/1751-7915.13994)
Supplement: Supplementary file 1 — Fig. S1. Nasal colonization of inoculated S. epidermidis was verified by 16S rRNA gene sequencing (A) S. epidermidis bacteria (ATCC 12228) (S. epi) (108 CFU) or nasal homogenates (0.2 g) (NH) of ICR mice were serially diluted (1:100‐1:105) and placed on a 500 µg/mL streptomycin supplemented TSB agar plate at 37°C for 24 h. S. epidermidis, but not endogenous bacteria in nasal cavities, can grow on streptomycin supplemented TSB agar plates. (B) ICR mice were intranasally inoculated 108 CFU/ 20 µL S. epidermidis every day for 3 days. Nasal homogenates (0.1 g/ 100 μL) were placed on a streptomycin supplemented TSB agar plate for 24 h. One of colonies (arrow) were selected for bacterial identification by 16S rRNA gene sequencing. (C) The nucleotide sequence of 16S rRNA gene of the selected colony shared 99% identity to S. epidermidis CLC‐M16. Fig. S2. Nasal administration of LCC did not affect the NPP‐induced IL‐6. LCC (+LCC) or PBS (‐LCC) was administered to the nasal cavities of ICR mice 6 h before subsequent inoculation of recombinant NPP. The level of IL‐6 in BALF was measured by ELISA 6 h after NPP inoculation. The level of IL‐6 induced by LCC relative to that induced by PBS was expressed as fold change. Data are represented as mean ± SD, in triplicate, two‐tailed t‐tests. ns = non‐significant. Fig. S3. Treatment of mouse macrophage J774A.1 cells with recombinant NPP induced a higher IL‐6 level compared to treatment of cells with recombinant GFP. The NPP‐induced of IL‐6 was reduced by pre‐treatment of cells with butyric acid. The reduction was partially restored by treatment of GLPG‐0974, a Ffar2 antagonist. ELISA was used to quantify IL‐6. Dara represented as mean ± SD, in triplicate, two‐tailed t‐tests. P‐values of <0.001 (***). Fig. S4. A S. epidermidis strain isolated from human nasal cavities fermented LCC and produced electricity (A) A S. epidermidis K1 strain (107 CFU) isolated from human nasal cavities (Table S1) was incubated in rich media (M) with/without [file MBT2-15-1984-s001.docx]

**Supplemental information**

**Colonization of Nasal Cavities by *Staphylococcus epidermidis* Mitigates SARS-CoV-2 Nucleocapsid Phosphoprotein-induced Interleukin (IL)-6 in the Lung**

Ming-Shan Kao,^1^ Jen-Ho Yang**_,_**^1¶^ Arun Balasubramaniam,^1¶^ Supitchaya Traisaeng,^2^ Albert Jackson Yang,^1^ John Jackson Yang,^1^ Benjamin Prethiviraj Salamon,^1^ Deron R. Herr,^4^ and Chun-Ming Huang^1,3*^

^1^Department of Biomedical Sciences and Engineering, National Central University, Taoyuan, 32001, Taiwan

^2^Department of Life Sciences, National Central University, Taoyuan, 32001, Taiwan

^3^Department of Biomedical Science and Environment Biology, Kaohsiung Medical University, Kaohsiung, 80708, Taiwan

^4^ Department of Biology, San Diego State University, San Diego, CA 92182, USA

Running title: *S. epidermidis* mitigates SARS-CoV-2-induced IL-6

^¶^Equal contribution

^*^Correspondence: E-mail: [chunming@ncu.edu.tw](mailto:chunming@ncu.edu.tw) (C.M.H.)

**Fig. S1.**

**
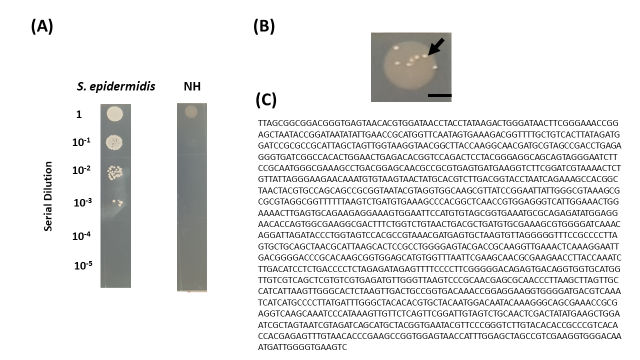
**

**Fig. S2.**


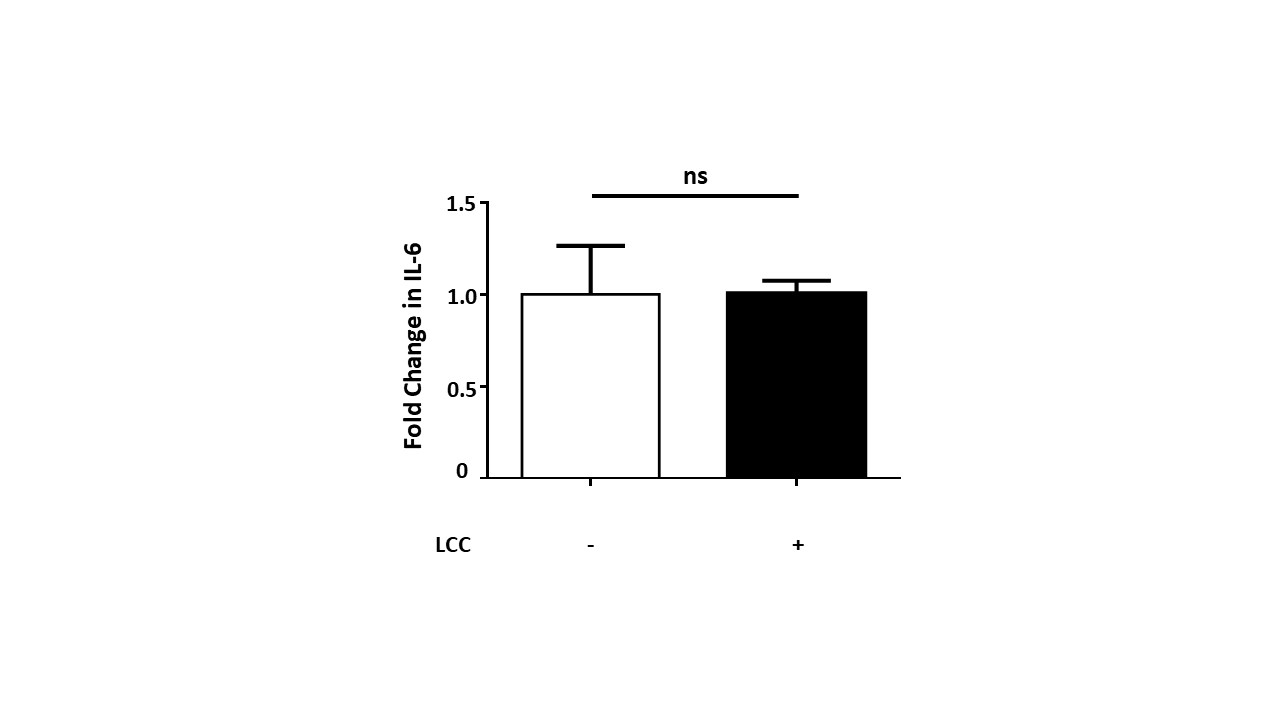


**Fig S3**


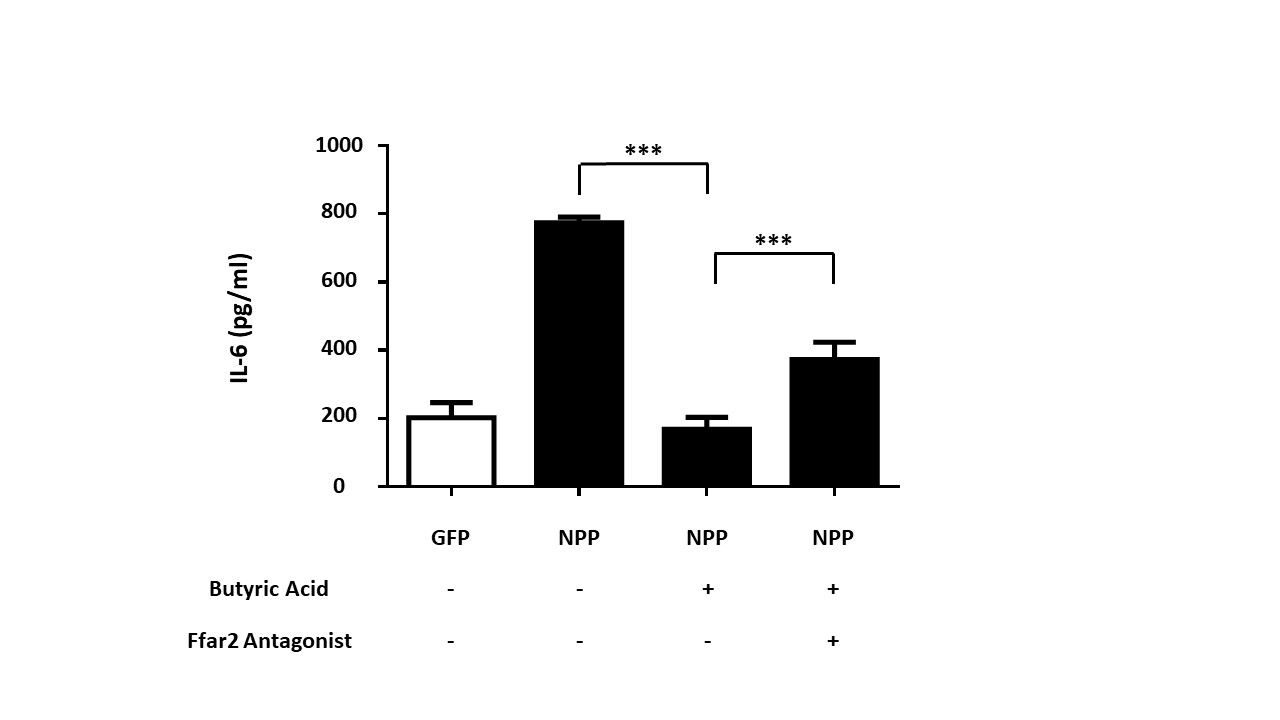


**Fig S4**

**
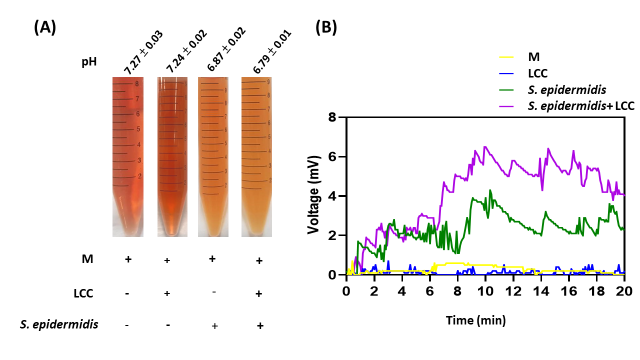
**

**Fig S5**

**
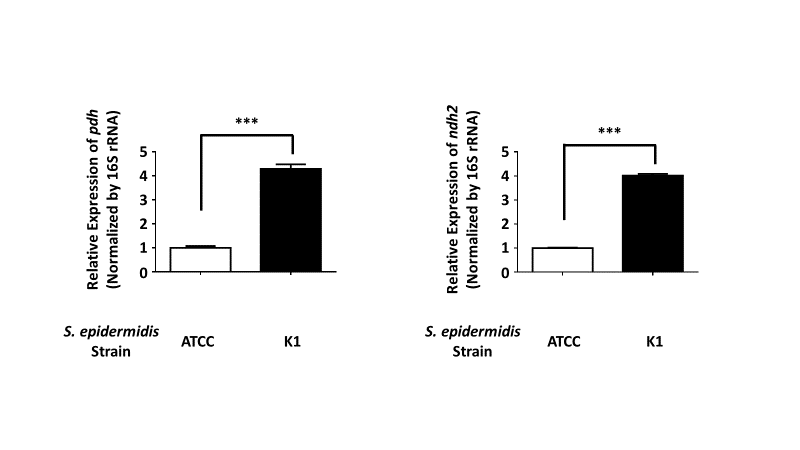
**

**Supplemental figure legends**

**Fig. S1.** Nasal colonization of inoculated *S. epidermidis* was verified by 16S rRNA gene sequencing (A) *S. epidermidis* bacteria (ATCC 12228) (*S. epi*) (10^8^ CFU) or nasal homogenates (0.2 g) (NH) of ICR mice were serially diluted (1:10^0^-1:10^5^) and placed on a 500 µg/mL streptomycin supplemented TSB agar plate at 37°C for 24 h. *S. epidermidis*, but not endogenous bacteria in nasal cavities, can grow on streptomycin supplemented TSB agar plates.

(B) ICR mice were intranasally inoculated 10^8^ CFU/ 20 µL *S. epidermidis* every day for 3 days. Nasal homogenates (0.1 g/ 100 μL) were placed on a streptomycin supplemented TSB agar plate for 24 h. One of colonies (arrow) were selected for bacterial identification by 16S rRNA gene sequencing.

(C) The nucleotide sequence of 16S rRNA gene of the selected colony shared 99% identity to *S. epidermidis* CLC-M16.

**Fig S2.** Nasal administration of LCC did not affect the NPP-induced IL-6. LCC (+LCC) or PBS (-LCC) was administered to the nasal cavities of ICR mice 6 h before subsequent inoculation of recombinant NPP. The level of IL-6 in BALF was measured by ELISA 6 h after NPP inoculation. The level of IL-6 induced by LCC relative to that induced by PBS was expressed as fold change. Data are represented as mean ± SD, in triplicate, two-tailed t-tests. ns = non-significant.

**Fig. S3**. Treatment of mouse macrophage J774A.1 cells with recombinant NPP induced a higher IL-6 level compared to treatment of cells with recombinant GFP. The NPP-induced of IL-6 was reduced by pre-treatment of cells with butyric acid. The reduction was partially restored by treatment of GLPG-0974, a Ffar2 antagonist. ELISA was used to quantify IL-6. Dara represented as mean ± SD, in triplicate, two-tailed t-tests. *P*-values of <0.001 (***).

**Fig. S4.** A *S. epidermidis* strain isolated from human nasal cavities fermented LCC and produced electricity (A) A *S. epidermidis* K1 strain (10^7^ CFU) isolated from human nasal cavities (Table S1) was incubated in rich media (M) with/without LCC for 12 h. Rich media alone and rich media plus LCC without *S. epidermidis* were included as controls. The pH values of media 12 h after fermentation were indicated. The color of phenol red in rich media changed from red-orange to yellow and reduction of pH values were used as indicators of bacterial fermentation.

(B) The *S. epidermidis* K1 strain (10^7^ CFU) with/without 2% LCC in rich media was pipetted on an anode. Pipetting media alone (M) or LCC alone acted as a control. The voltage difference (mV) between anode and cathode was monitored for 20 min.

**Fig. S5.** The expression levels of genes (*pdh* and *ndh2*) in the *S. epidermidis* ATCC 12228 and K1 strains. RT-qPCR was used to examine the relative expression of the *pdh* and *ndh2* genes which were normalized to 16S rRNA gene. Data shown represent the mean ± SD of experiments performed in triplicate, two-tailed t-tests. *P*-values of <0.001 (***).

**Supplemental table**

**Table S1.** 16S rRNA gene sequences of 11 bacterial colonies (K1, K2, H1, H2, and 1-7) isolated from human nasal cavities

| **Colony**  **Name** | **16S rRNA**  **Sequence** | **Species** | **%**  **Identify** |
| --- | --- | --- | --- |
| **K1** | GGCATGGCGGCGTGCTATACATGCAGTCGAGCGAACAGACGAGGAGCTTGCTCCTCTGACGTTAGCGGCGGACGGGTGAGTAACACGTGGATAACCTACCTATAAGACTGGGATAACTTCGGGAAACCGGAGCTAATACCGGATAATATATTGAACCGCATGGTTCAATAGTGAAAGACGGTTTTGCTGTCACTTATAGATGGATCCGCGCCGCATTAGCTAGTTGGTAAGGTAACGGCTTACCAAGGCAACGATGCGTAGCCGACCTGAGAGGGTGATCGGCCACACTGGAACTGAGACACGGTCCAGACTCCTACGGGAGGCAGCAGTAGGGAATCTTCCGCAATGGGCGAAAGCCTGACGGAGCAACGCCGCGTGAGTGATGAAGGTCTTCGGATCGTAAAACTCTGTTATTAGGGAAGAACAAATGTGTAAGTAACTATGCACGTCTTGACGGTACCTAATCAGAAAGCCACGGCTAACTACGTGCCAGCAGCGGGCCGGTA | *S. epidermidis* | 100 |
| **K2** | ATGGAGTGCGGCGTGCTATACATGCAGTCGAGCGAACAGACGAGGAGCTTGCTCCTCTGACGTTAGCGGCGGACGGGTGAGTAACACGTGGATAACCTACCTATAAGACTGGGATAACTTCGGGAAACCGGAGCTAATACCGGATAATATATTGAACCGCATGGTTCAATAGTGAAAGACGGTTTTGCTGTCACTTATAGATGGATCCGCGCCGCATTAGCTAGTTGGTAAGGTAACGGCTTACCAAGGCAACGATGCGTAGCCGACCTGAGAGGGTGATCGGCCACACTGGAACTGAGACACGGTCCAGACTCCTACGGGAGGCAGCAGTAGGGAATCTTCCGCAATGGGCGAAAGCCTGACGGAGCAACGCCGCGTGAGTGATGAAGGTCTTCGGATCGTAAAACTCTGTTATTAGGGAAGAACAAATGTGTAAGTAACTATGCACGTCTTGACGGTACCTAATCAGAAAGCCACGGCTAACTACGTGCCAGCAGCGGCCGGTAAT | *S. epidermidis* | 99.6 |
| **H1** | GGGCATGCGGCGTGCTATACATGCAGTCGAGCGAACAGACGAGGAGCTTGCTCCTCTGACGTTAGCGGCGGACGGGTGAGTAACACGTGGATAACCTACCTATAAGACTGGGATAACTTCGGGAAACCGGAGCTAATACCGGATAATATATTGAACCGCATGGTTCAATAGTGAAAGACGGTTTTGCTGTCACTTATAGATGGATCCGCGCCGCATTAGCTAGTTGGTAAGGTAACGGCTTACCAAGGCAACGATGCGTAGCCGACCTGAGAGGGTGATCGGCCACACTGGAACTGAGACACGGTCCAGACTCCTACGGGAGGCAGCAGTAGGGAATCTTCCGCAATGGGCGAAAGCCTGACGGAGCAACGCCGCGTGAGTGATGAAGGTCTTCGGATCGTAAAACTCTGTTATTAGGGAAGAACAAATGTGTAAGTAACTATGCACGTCTTGACGGTACCTAATCAGAAAGCCACGGCTAACTACGTGCCAGCAGGGCCGGGTA | *S. epidermidis* | 100 |
| **H2** | GGATGCGGGTGCTATACATGCAGTCGAGCGAACAGACGAGGAGCTTGCTCCTCTGACGTTAGCGGCGGACGGGTGAGTAACACGTGGATAACCTACCTATAAGACTGGGATAACTTCGGGAAACCGGAGCTAATACCGGATAATATATTGAACCGCATGGTTCAATAGTGAAAGACGGTTTTGCTGTCACTTATAGATGGATCCGCGCCGCATTAGCTAGTTGGTAAGGTAACGGCTTACCAAGGCAACGATGCGTAGCCGACCTGAGAGGGTGATCGGCCACACTGGAACTGAGACACGGTCCAGACTCCTACGGGAGGCAGCAGTAGGGAATCTTCCGCAATGGGCGAAAGCCTGACGGAGCAACGCCGCGTGAGTGATGAAGGTCTTCGGATCGTAAAACTCTGTTATTAGGGAAGAACAAATGTGTAAGTAACTATGCACGTCTTGACGGTACCTAATCAGAAAGCCACGGCTAACTACGTGCCAGCAGGGGCCCGGTAA | *S. epidermidis* | 100 |
| **1** | ATGAGTGCGGCGTGCTATACATGCAGTCGAGCGGACAGAAGGGAGCTTGCTCCCGGATGTTAGCGGCGGACGGGTGAGTAACACGTGGGTAACCTGCCTGTAAGACTGGGATAACTCCGGGAAACCGGAGCTAATACCGGATAGTTCCTTGAACCGCATGGTTCAAGGATGAAAGACGGTTTCGGCTGTCACTTACAGATGGACCCGCGGCGCATTAGCTAGTTGGTGGGGTAATGGCTCACCAAGGCGACGATGCGTAGCCGACCTGAGAGGGTGATCGGCCACACTGGGACTGAGACACGGCCCAGACTCCTACGGGAGGCAGCAGTAGGGAATCTTCCGCAATGGACGAAAGTCTGACGGAGCAACGCCGCGTGAGTGATGAAGGTTTTCGGATCGTAAAGCTCTGTTGTTAGGGAAGAACAAGTGCGAGAGTAACTGCTCGCACCTTGACGGTACCTAACCAGAAAGCCACGGCTAACTACGTGCCAGCAGCCGCGGTAA | *B. pumilus* | 99.8 |
| **2** | GTGCCTGCGGCTGCTATACATGCAGTCGAGCGAACAGACGAGGAGCTTGCTCCTCTGACGTTAGCGGCGGACGGGTGAGTAACACGTGGATAACCTACCTATAAGACTGGGATAACTTCGGGAAACCGGAGCTAATACCGGATAATATATTGAACCGCATGGTTCAATAGTGAAAGACGGTTTTGCTGTCACTTATAGATGGATCCGCGCCGCATTAGCTAGTTGGTAAGGTAACGGCTTACCAAGGCAACGATGCGTAGCCGACCTGAGAGGGTGATCGGCCACACTGGAACTGAGACACGGTCCAGACTCCTACGGGAGGCAGCAGTAGGGAATCTTCCGCAATGGGCGAAAGCCTGACGGAGCAACGCCGCGTGAGTGATGAAGGTCTTCGGATCGTAAAACTCTGTTATTAGGGAAGAACAAATGTGTAAGTAACTATGCACGTCTTGACGGTACCTAATCAGAAAGCCACGGCTAACTACGTGCCAGCAGCGGGCCGGTA | *S. epidermidis* | 100 |
| **3** | TTGGAGTGCGGCGTGCTATACATGCAGTCGAGCGCAGGAAACCAGATGACCCCTTCGGGGTGATTCTGGTGGAATGAGCGGCGGACGGGTGAGTAACACGTGGGCAACCTGCCTGTAAGACTGGGATAACTTCGGGAAACCGGAGCTAATACCGGATAGTATTTCCTTTCTCCTGATTGGAAATGGAAAGACGGTTTCGGCTGTCACTTACAGATGGGCCCGCGGTGCATTAGCTAGTTGGTGGGGTAATGGCCCACCAAGGCGACGATGCATAGCCGACCTGAGAGGGTGATCGGCCACACTGGGACTGAGACACGGCCCAGACTCCTACGGGAGGCAGCAGTAGGGAATCTTCCGCAATGGACGAAAGTCTGACGGAGCAACGCCGCGTGAGCGATGAAGGCCTTCGGGTCGTAAAGCTCTGTTGTTAGGGAAGAACAAGTACGAGAGTAACTGCTCGTACCTTGACGGTACCTAACCAGAAAGCCCCGGCTAACTACGTGCCAGCAGCCGCCGGTA | *T. goriensis* | 99.8 |
| **4** | CTGGATGCGCTGCTATACATGCAGTCGAGCGAACAGACGAGGAGCTTGCTCCTCTGACGTTAGCGGCGGACGGGTGAGTAACACGTGGATAACCTACCTATAAGACTGGGATAACTTCGGGAAACCGGAGCTAATACCGGATAATATATTGAACCGCATGGTTCAATAGTGAAAGACGGTTTTGCTGTCACTTATAGATGGATCCGCGCCGCATTAGCTAGTTGGTAAGGTAACGGCTTACCAAGGCAACGATGCGTAGCCGACCTGAGAGGGTGATCGGCCACACTGGAACTGAGACACGGTCCAGACTCCTACGGGAGGCAGCAGTAGGGAATCTTCCGCAATGGGCGAAAGCCTGACGGAGCAACGCCGCGTGAGTGATGAAGGTCTTCGGATCGTAAAACTCTGTTATTAGGGAAGAACAAATGTGTAAGTAACTATGCACGTCTTGACGGTACCTAATCAGAAAGCCACGGCTAACTACGTGCCAGCAGGGGCCGGTA | *S. epidermidis* | 99.8 |
| **5** | GGGCTGGATGCGCGTGCTATACATGCAGTCGAGCGAACAGACGAGGAGCTTGCTCCTCTGACGTTAGCGGCGGACGGGTGAGTAACACGTGGATAACCTACCTATAAGACTGGGATAACTTCGGGAAACCGGAGCTAATACCGGATAATATATTGAACCGCATGGTTCAATAGTGAAAGACGGTTTTGCTGTCACTTATAGATGGATCCGCGCCGCATTAGCTAGTTGGTAAGGTAACGGCTTACCAAGGCAACGATGCGTAGCCGACCTGAGAGGGTGATCGGCCACACTGGAACTGAGACACGGTCCAGACTCCTACGGGAGGCAGCAGTAGGGAATCTTCCGCAATGGGCGAAAGCCTGACGGAGCAACGCCGCGTGAGTGATGAAGGTCTTCGGATCGTAAAACTCTGTTATTAGGGAAGAACAAATGTGTAAGTAACTATGCACGTCTTGACGGTACCTAATCAGAAAGCCACGGCTAACTACGTGCCAGCAGCGGGCCGGTAA | *S. epidermidis* | 99.8 |
| **6** | GTCAGTGCGGCGTGCTTACACATGCAGTCGAACGCTGAGGCTTGGTGCTTGCACTGGGTGGATGAGTGGCGAACGGGTGAGTAATACGTGAGTAACCTGCCCTTGACTCTGGGATAAGCCTGGGAAACTGGGTCTAATACTGGATATGACCTCCTGTCGCATGGTGGGGGGTGGAAAGGGTTTGTACTGGTTGTGGATGGGCTCACGGCCTATCAGCTTGTTGGTGGGGTAATGGCCTACCAAGGCGACGACGGGTAGCCGGCCTGAGAGGGTGACCGGCCACACTGGGACTGAGACACGGCCCAGACTCCTACGGGAGGCAGCAGTGGGGAATATTGCACAATGGGCGCAAGCCTGATGCAGCGACGCCGCGTGAGGGATGACGGCCTTCGGGTTGTAAACCTCTTTCAGCACGGAAGAAGCGAAAGTGACGGTACGTGCAGAAGAAGCGCCGGCTAACTACGTGCCAGCAGCGGCGGTA | *K. indica* | 99.16 |
| **7** | CAGAATTTGCGGCGTGCTTTACCATGCAGTCGAACGGAAAGGCCCTGCTTGCAGGGTACTCGAGTGGCGAACGGGTGAGTAACACGTGGGTGATCTGCCCTGCACTTCGGGATAAGCTTGGGAAACTGGGTCTAATACCGGATAGGAGCCATCTTTAGTGTGGTGGTTGGAAAGTTTTTTCGGTGTAGGATGAGCTCGCGGCCTATCAGCTTGTTGGTGGGGTAATGGCCTACCAAGGCGGCGACGGGTAGCCGGCCTGAGAGGGTGTACGGCCACATTGGGACTGAGATACGGCCCAGACTCCTACGGGAGGCAGCAGTGGGGAATATTGCACAATGGGCGCAAGCCTGATGCAGCGACGCCGCGTGGGGGATGAAGGCCTTCGGGTTGTAAACTCCTTTCGCTAGGGACGAAGCTTTTTGTGACGGTACCTAGATAAGAAGCACCGGCTAACTACGTGCCAGCAGCCGCGGTAA | *C. segmentosum* | 99.36 |

**Table S2.** The designed primers for EET related genes

| **Gene** | **Primer Sequence** |
| --- | --- |
| ***pdh*** | Forward 5’ CTTCTACTGATGTCGTTAATGCTTCTG 3’  Reverse 5’ GCAATTGCTTTGCGCATTG 3’ |
| ***ndh2*** | Forward 5’ TCTGCTAGTTGAGCACTGGG 3’  Reverse 5’ TGGACAGCAGGCATACAACC 3’ |
| **16S rRNA** | Forward 5’ ATGCACGTCTTGACGGTACCT 3’  Reverse 5’ TCCATGGCAGTTCTGCACGTA 3’ |

**Supplemental methods**

*Nasal inoculation of LCC*

LCC (2% in 20 μL PBS) was administered to the nasal cavities of ICR mice for 6 h. Inoculation of PBS (20 μL) as a control. After administration of LCC or PBS, recombinant NPP (50 μg in 20μL PBS) was subsequently inoculated into nasal cavities for 6 h. The level of IL-6 in BALF was quantified by ELISA using a Quantikine mouse IL-6 set (R&D Systems).

*Identification of bacteria in human nasal cavities*

The nasal swab samples were streaked by plating on a TSB (Sigma) agar plate at 37°C for 24 h. Eleven colonies were selected and cultured in 10 mL of TSB media at 37°C for 24 h. The genomic DNA was extracted from the selected colonies using an Easy Pure Genomic DNA Spin Kit (Bioman, New Taipei, Taiwan). Sequence analysis of 16S rRNA genes was performed for bacterial identification using the 16S rRNA 27F and 534R primers for PCR. The sequences were employed for species identification by the Basic Local Alignment Search Tool (BLAST). An identified *S. epidermidis* K1 strain was used to detect fermentation and electricity in the presence or absence of LCC.

*Macrophage cultures and treatments*

The mouse macrophage J774A.1 cell lines were incubated in a 96-well plate (1 x 10^5^ cells/ 200 μL) in Dulbecco’s modified essential media (Gibco-BRL, Grand Island, NY, USA) with 10% (v/v) fetal bovine serum (FBS) (Irvine Scientific, Santa Ana, CA, USA), and 100 diluted Pen-Strep-Ampho. B solution (Biological Industries, Kibbutz Beit HaEmek, Israel) at 37°C for 2 days. After removing the supernatants, cells were subsequently treated with 0.1 μM GLPG-0974 or the same volume of Dimethyl sulfoxide (DMSO) in the absence of FBS for 6 h. Butyric acid (1 mM) or PBS was added 1 h before treating with 25 μg recombinant NPP. After treatment of recombinant NPP for 12 h, the IL-6 in the cell media was measured by ELISA.

*LCC fermentation of S. epidermidis K1 strain*

To induce fermentation, *S. epidermidis* K1 strain (10^7^ CFU/mL) was incubated in rich media in the absence or presence of 2% LCC at 37°C for 12 h. Rich media or rich media plus LCC without bacteria were included as controls. Phenol red [0.001% (w/v), Sigma] in rich media with LCC served as an indicator of fermentation, converting from red-orange to yellow when fermentation occurred (Kao, et al., 2017). The pH value of fermentation media was measured.

*RT-qPCR*

RT-qPCR was used to analyze the expression of genes encoding *pdh* and *ndh2* in *S. epidermidis* ATCC 12228 and K1 strains. RNA (1 ng) was converted to cDNA using an iScript cDNA Synthesis Kit (Bio-Rad, Hercules, CA, USA). The cDNA was served as a template in StepOnePlus RT PCR System (Thermo Fisher Scientific, Waltham, MA, USA), which was executed using Power SYBR Green and PCR Master Mix (Thermo Fisher Scientific). The primer-Blast tool (https://blast.ncbi.nlm.nih.gov/Blast.cgi/; Rockville Pike, Bethesda MD, USA) from the National Center for Biotechnology Information (NCBI) was employed for designing all primers. One step RT-PCR reaction was fixed for 40 cycles as follows: 95°C for 10 min followed by 95°C for 15 s, 60°C for 60 s, and 72°C for 30 s. Gene expression was normalized with the 16S rRNA gene. The cycle threshold (2−ΔΔCt) was implemented to analyze the relative expression of genes. The designed primers for all genes were shown in Table S2.

**Supplemental references**

Kao, M.S., Huang, S., Chang, W.L., Hsieh, M.F., Huang, C.J., Gallo, R.L., and Huang, C.M. (2017) Microbiome precision editing: Using PEG as a selective fermentation initiator against methicillin-resistant Staphylococcus aureus, *Biotechnol J* **12**.
